# Supplementary figures and images for: Evolution of CDK1 Paralog Specializations in a Lineage With Fast Developing Planktonic Embryos
Source: Front Cell Dev Biol. 2022 Jan 28;9:770939. doi: 10.3389/fcell.2021.770939 (PMC8832800; doi:10.3389/fcell.2021.770939)

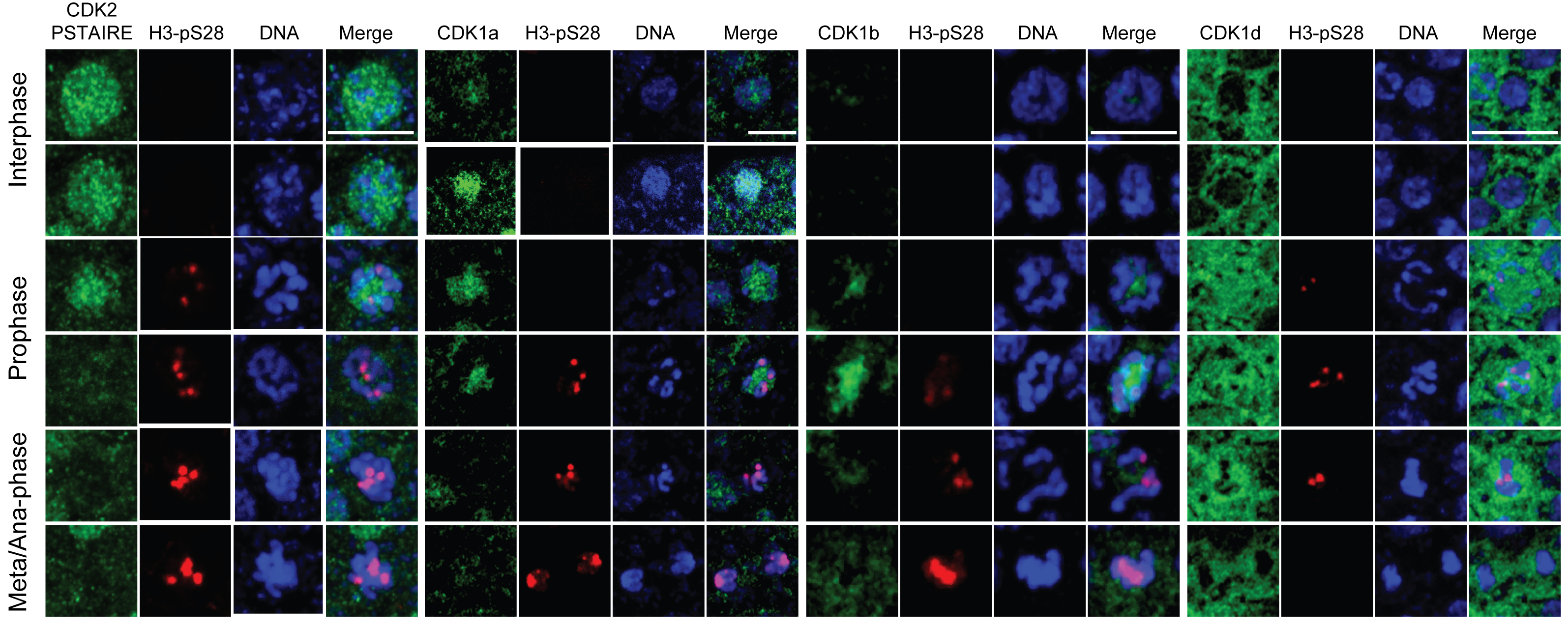

Supplement: Supplementary file 1 [file Image6.TIF]

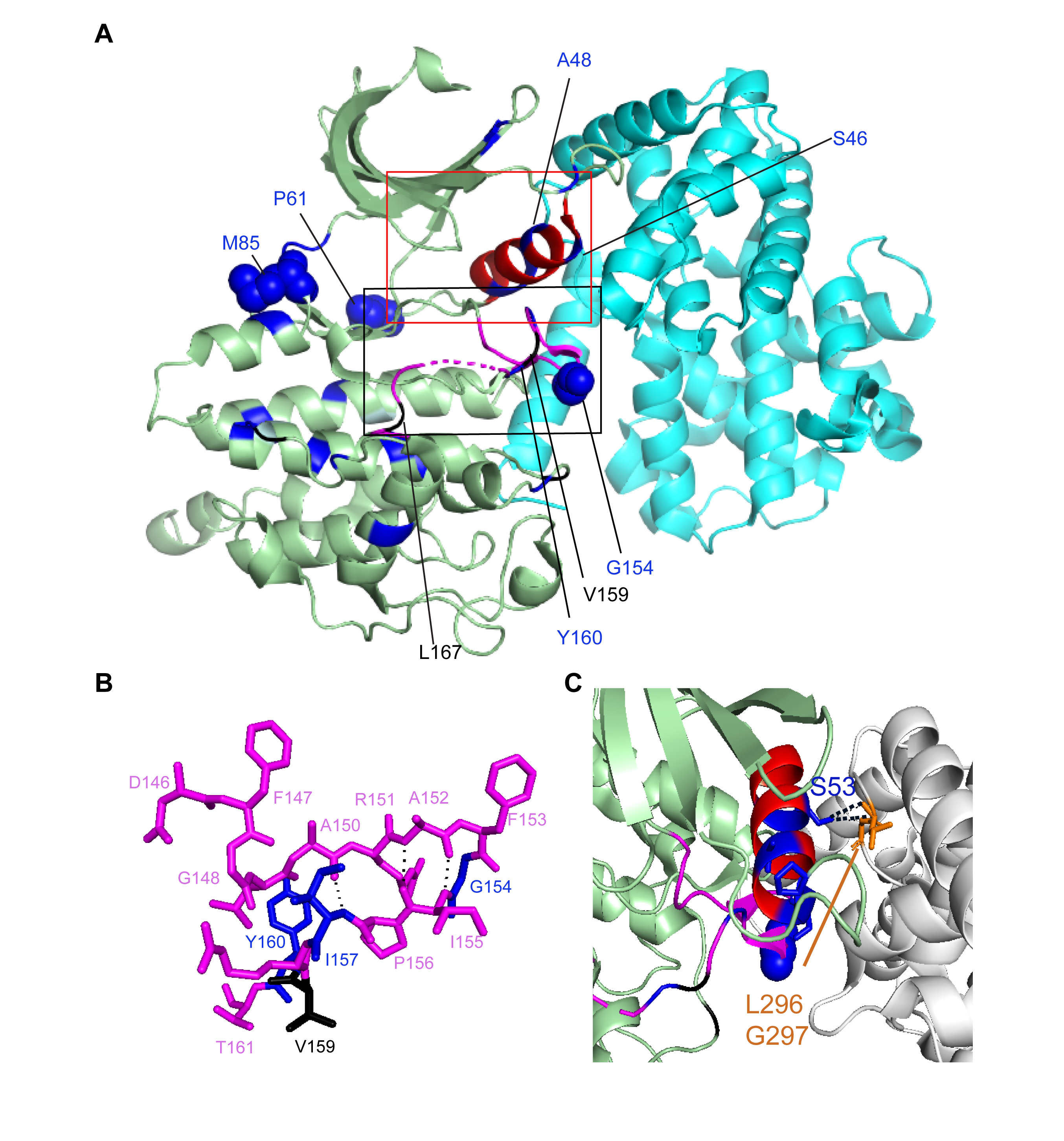

Supplement: Supplementary file 2 [file Image3.TIF]

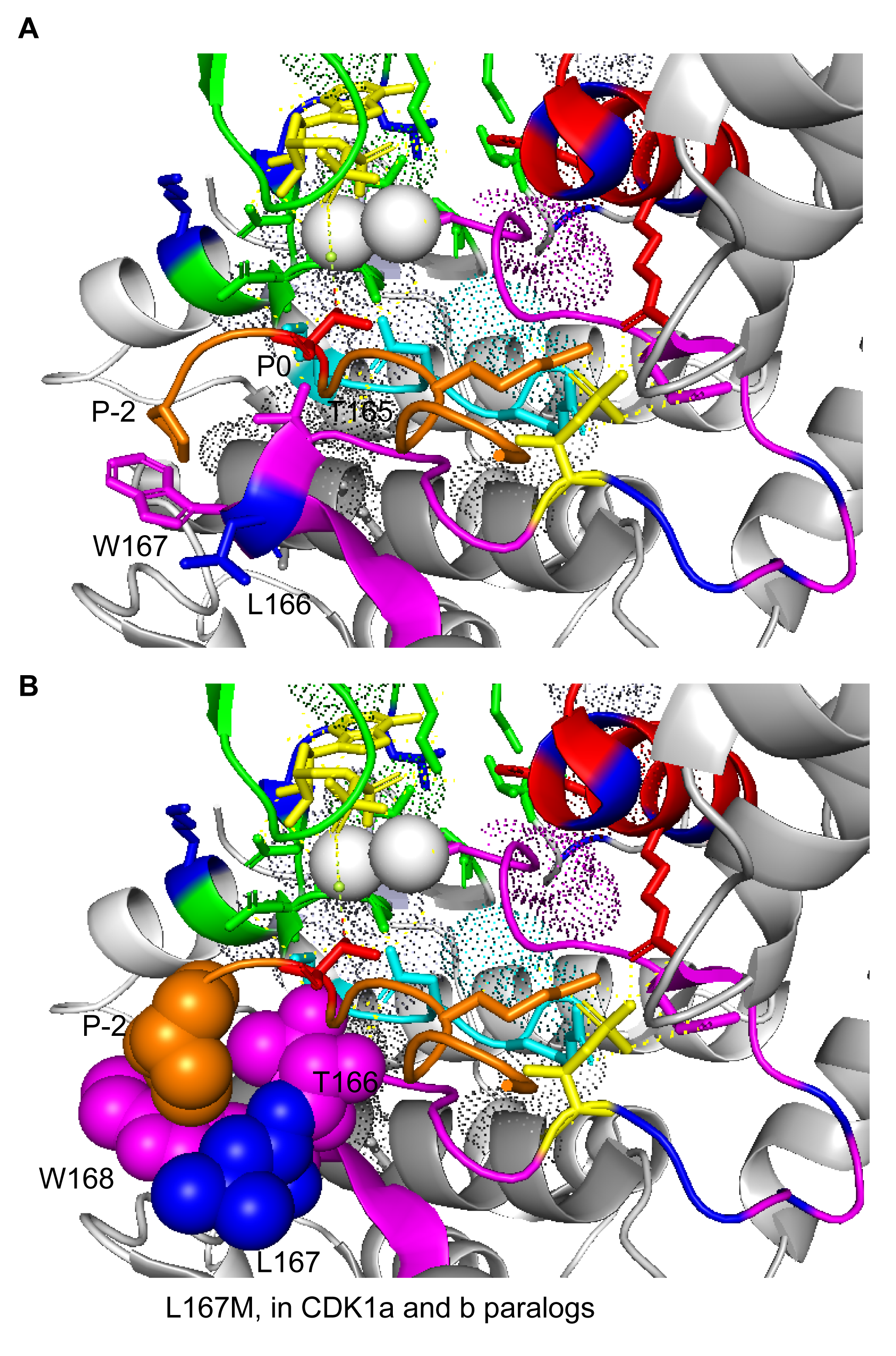

Supplement: Supplementary file 3 [file Image4.TIF]

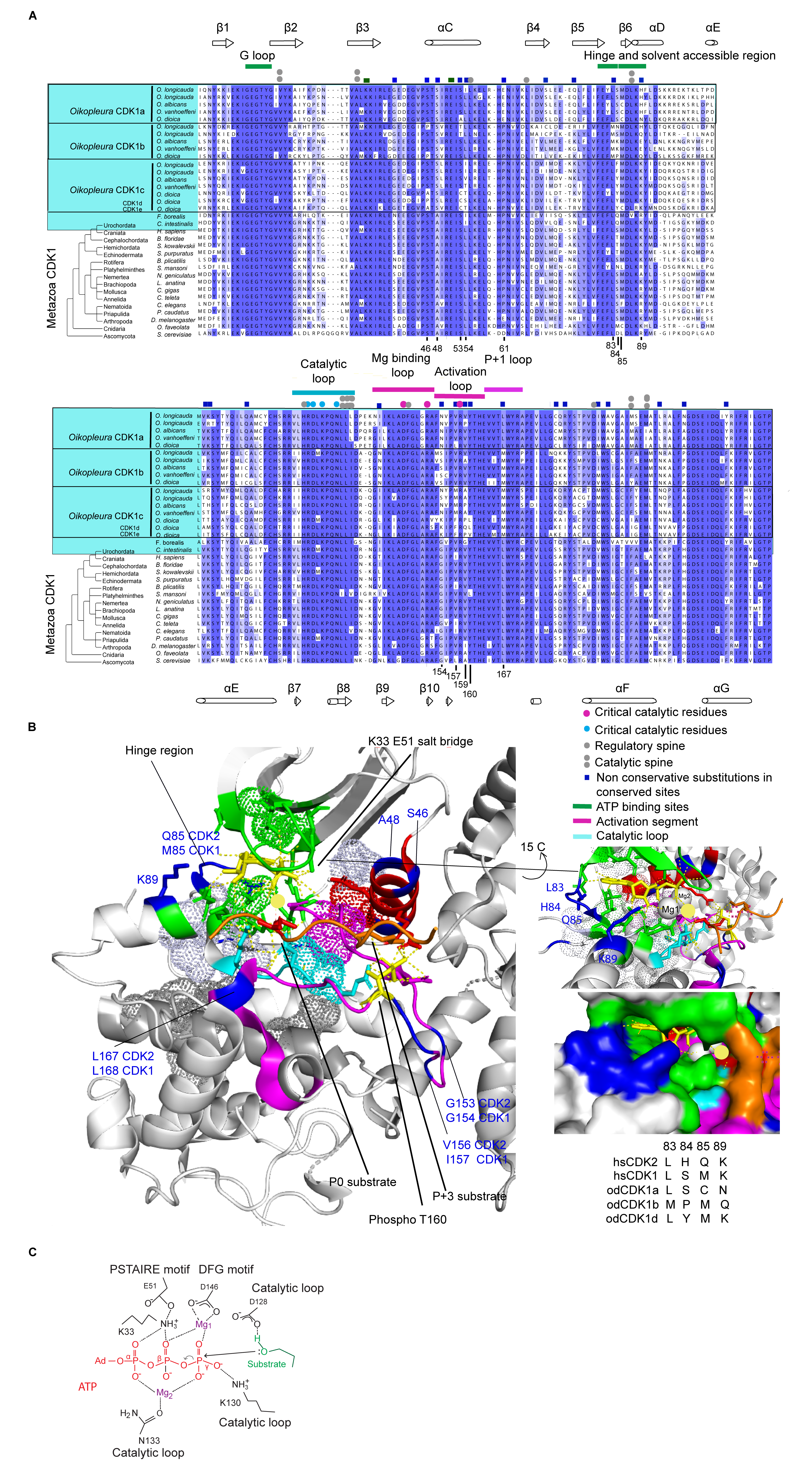

Supplement: Supplementary file 4 [file Image2.TIF]

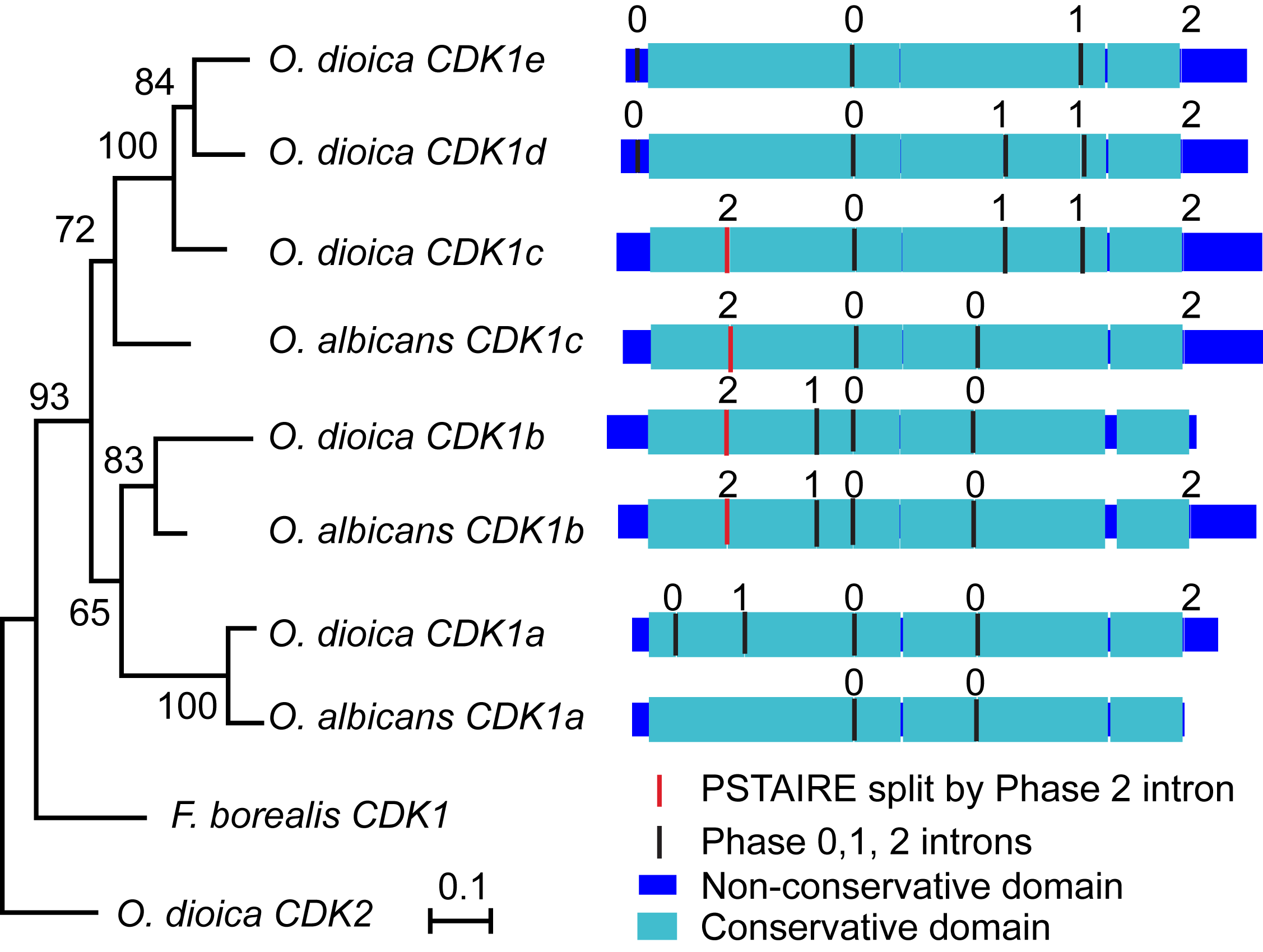

Supplement: Supplementary file 5 [file Image1.TIF]

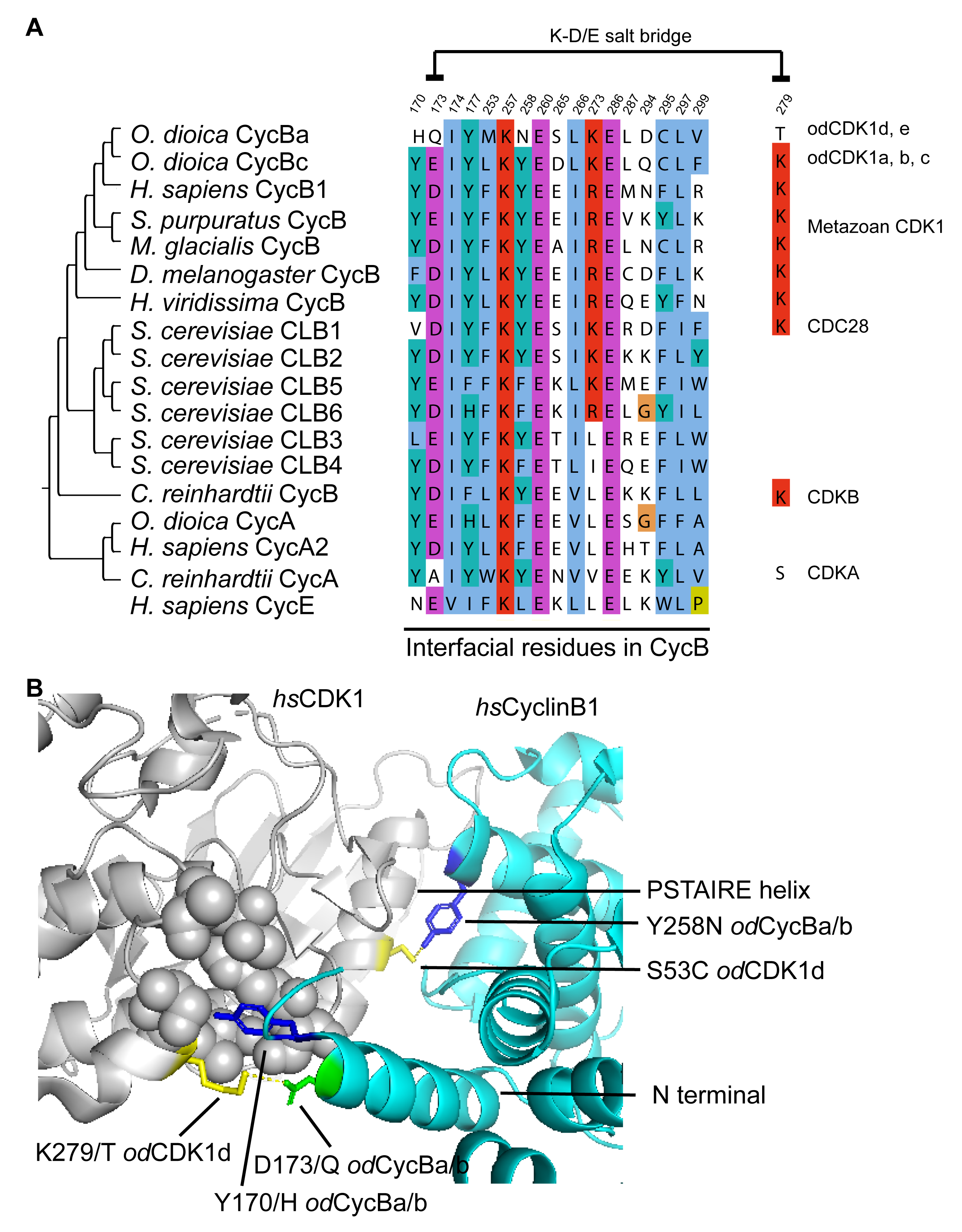

Supplement: Supplementary file 6 [file Image7.TIF]

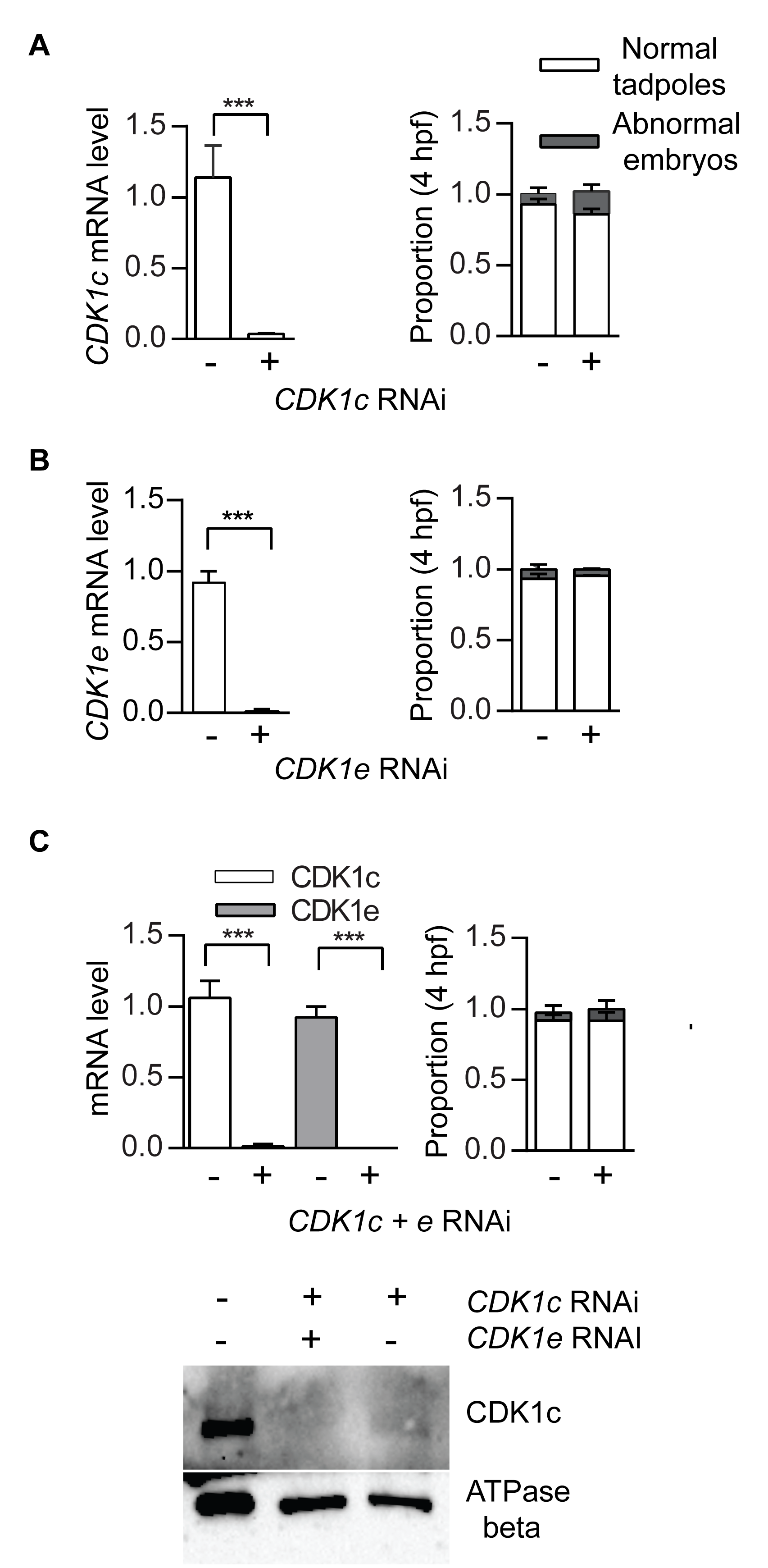

Supplement: Supplementary file 8 [file Image5.TIF]
