# Supplementary material for: Evolution of CDK1 Paralog Specializations in a Lineage With Fast Developing Planktonic Embryos
Source: Front Cell Dev Biol. 2022 Jan 28;9:770939. doi: 10.3389/fcell.2021.770939 (PMC8832800; doi:10.3389/fcell.2021.770939)
Supplement: Supplementary file 7 [file DataSheet1.PDF]

SUPPLEMENTARY INFORMATION

Evolution of multiple CDK1 paralog specializations in a lineage with fast developing planktonic embryos

Xiaofei Ma[1*+], Jan Inge Øvrebø[1,2#], Eric M Thompson[1,2 *3]

[1]Sars International Centre, and [2]Dept. of Biological Sciences, University of Bergen, Bergen, Norway

[#]Present address: Huntsman Cancer Institute, University of Utah, USA

+ Current address: College of Life Sciences, Northwest Normal University, China

[3]lead correspondence

*Correspondence to: Eric.Thompson@uib.no, ORCID 0000-0002-3756-9036
Xiaofei.ma@uib.no

SUPPLEMENTARY METHODS

Test of Selection

Branch-sites tests of selection are phylogeny-based tests that require the *a priori* identification of a foreground branch that is hypothesized to be evolving at a different rate than the background branches in the rest of the tree. Codons are alignment with PRANK, gaps in alignment were deleted. We first set a series of nested models specifying particular branches as foreground branches: the lineage subtending the *CDK1a* clade, the lineage subtending the *CDK1b* clade, the lineage subtending the *CDK1c* clade. Using a likelihood ratio test, we then tested the hypothesis, that a model which included one class of sites with $\omega > 1$, indicating positive selection, was superior to a model where $\omega = 1$. We estimated codon frequencies on the basis of observed nucleotide frequencies at each of the three codon positions, as a procedure to reduce false positives due to potential nucleotide biases. We also used the empirical Bayes method as implemented in PAML 4 (Yang 2007) to identify the positively selected sites along the 3 branches. The synonymous (silent) and non-synonymous (non-silent) nucleotide substitution rates of each branch were also estimated by the modified Nei–Gojobori method (Zhang et al. 1998), based on tree topology inferred by maximum likelihood (ML) using MEGA (Kumar et al. 2016).

Homology Modeling

Active and inactive hsCDK1 was predicted with I-TASSER (Yang et al. 2015), using the hsCDK1-cyclin B crystal structure (Brown et al. 2015). 3D models were visualized using Pymol (DeLano 2002). Based on alignment of odCDK1 paralogs and hsCDK1, nonconservative variations between odCDK1s and hsCDK1 were colored in the models.

Recombinant protein expression in insect cells

Coding regions of Cyclin Ba and OdCdk1d were cloned into pCR™8/GW/TOPO and recombined into pDEST™10 (N-terminal His_6-TEV fusion tag) or pDEST™20 (N-terminal GST fusion tag), according to manufacturer's (Thermofisher Scientific) instructions. The EMBL Protein Expression and Purification Core Facility performed protein expression and purification. The pDEST10_Cyclin Ba and pDEST20_OdCdk1d plasmid DNA were used for transposition into *E. coli* DH10EMBacY cells. Bacmid DNA was isolated from a 3 ml overnight culture of positive (white) colonies according to the MultiBac protocol (Nie et al. 2013). The bacmid DNA was mixed with X-tremeGENE transfection reagent and Sf-900 III SFM medium and the mixture was added to Sf21 insect cells. After 3 days, the V_0 virus was harvested and used to infect a larger Sf21 culture for V_1 virus generation. For co-expression of His_6-tagged Cyclin Ba and GST-tagged OdCdk1d, 500 ml Sf21 insect cells was co-infected with 2 ml Cyclin Ba V_1 virus and 2 ml OdCdk1d V_1 virus and cultured for 72 h at 27°C. Cells were harvested by centrifugation and resuspended in running buffer (50 mM Tris pH 8.0, 250 mM NaCl and 20 mM imidazole) supplemented with benzonase and cOmplete EDTA-free protease inhibitors. Cells were lysed using a Dounce homogenizer and the lysate was cleared by centrifugation (10000 x *g*, 30 min,

4°C). The cleared lysate was incubated with 200 µl Ni-NTA (Qiagen) beads for 2 h at 4°C. After incubation, the mixture was transferred to an empty PD10 column, the beads were washed with running buffer and then eluted in 200 µl fractions with running buffer supplemented with 300 mM imidazole. All fractions were analysed by SDS-PAGE and the fractions containing the Cyclin Ba–OdCdk1d complex were concentrated, flash-frozen in liquid nitrogen and stored at -80°C until further use.

DsRNA sequences used for RNAi

CDK1a dsRNA

ATGACGAAGGCGTACCAAGCACTAGTATAAGAGAGATCGCCTTATTGAAAGAGCTCAAG
CACGAAAATATTGTCAAACTTATCGATGTGTCGCTGGACGAAGAGCAACTCTTTCTCATT
TTCGAGTTTCTTTCGTGCGATCTAAAAAACTATCTCGATAAGCAACGAAGAGCGAAAAA
ACGTCTTGATCAGATTACTGTCAAGTCTTATACATTCCAAATACTACAGGCACTTTCGTTT
TGTCATTCAAGAAGAGTTCTACATCGAGATCTAAAGCCTCAAAATCTGCTTATATCTCCA
GAAACCGGGATACTAAAACTAGCGGATTTCGGCCTTGGAAGAGCGTTTAACATTCCACT
GCGGGTATACACTCATGAAGTCGTTACCCTTTGGTATCGAGCTCCAGAAGTCCTTCTCGG
CTGTTTGAGATACTCTATTCCAATTGATATGTGGGCTGTCGGAGCGATCATGGCCGAAAT
AGCAACGCTCAGAGCACTTTTCGCTGGCGACTCAGAGATCGACCAGCTTTACAGAATCTT
CCGGATTTTAGGAACGCCGTCGAATAAGATTTGGAAGGGCGTCGAGAATTTCAAGGACT

CDK1b dsRNA

TTTCGCCTTGGAGACGAGGAAGAAGGAATTCCACCAACAAGCGTTCGCGAAATCTCACT
GCTAAAAGAATTGAAACACCCGAACATTGTCGATCTGATCACGATTTTGGTCGAGAAAG
AAAAAATCTATCTCGTCTTTGAGTTTATGCCAATGGATCTCAAACAATATCTCGACTCGC
TTAAAAGCAGTGGAAAATTCATGCGAGAAAAACTCGTCCGATCCTACATGTTTCAGCTC
ATTTGTGGTCTTTCATTTTGTCATTCTCGTCGAATCCTGCATCGAGACTTGAAGCCTCAAA
ATCTGTTGATCGACGAATCTGGAAACATCAAACTTGCAGATTTCGGACTTGCTCGAGCAG
TTTCGATCCCAGTTCGCGTTTACACTCACGAAATCATCACCATGTGGTATCGTGCTCCCG
AAATTCTTCTCGGACA

CDK1c dsRNA

TTCTCGGAACACCGACCGAAGAAACCTGGCCGGGAGTCTCCCAGCTTTCAGAGTTCAAT
CTAAACTTCCCGATCTTTCCACGCGGAACATTTCCAAACCCACAGCGCTTCAAACTTTCC
GCGAGTGCTGTAGATCTCGTTCATAAATTTCTCGCTTTTGATCCGGCGAAGAGACTCACT
GCAAAGGCAGCTCTAAAGCATCCATTTTTCGACAGGCTTAATAAAACGCTCTTTCCGGGA
AATAAATGCCCGGCTGTACCTCACTACGACAAAGAGGCGTCAAAAGCTGCGGCGATCTT
GAATCTTCCTCT

CDK1d dsRNA

GGCATTCCGCCTACCAGTCTGCGTGAGATCTGCTCACTCAAGGAACTCGCTCACCCGAAC
ATCGTCAAGCTGATCGATGTCATTCTCGAGAAAACTAGAGTCTACCTTGTCTTTGAATTC
TTATACATGGACCTGAAGAAGTACATTGATGATCAAAAGGAGCAAGAAAGTAGGATCG
ATAGGAGCTTGGCGATGAG

CDK1e dsRNA

TACGCATGTCCGGTTGATTGTTGGTCGCTTGGTGCAATCATCGGTGAAATGTTAACGAAC
GTGGCTGTCTTCCCGGGCGATTCTGAGATTGACCAGCTTTTCAAGATATTCAGAGTGCTT
GGGACGCCAAATGAATCGATATGGCCAGGAGTTTCGGAACTTAAAGAATTCAGCCTGAA
TTTCCCGATCTTCCCGAAAGGAGAAATCCCGAACCCGGATCGCTTTGATATACCTTCCAA
AGCACGCGACTTGGTACTAAAGATGCTAACATATGACCCAGTTAAGAGAATGACAACAG
CTCAGGCTCTTTGTCATCCTTATTTCGACAGGCTGGAGAAGTCGATATTTCCCAGCAAGG
AATATCCAGCTGTCCCTGCT

CDK2 dsRNA

TAAAAACAGAGCAACAGGCAGACTTGTTGCACTCAAGAAAATCAGACTGGAAACAGAA
TCCGAAGGCGTTCCGTCTACGGCGATACGTGAGATTTCTTTATTGAAGGAACTCGATCAT
CCGAATGTCGTTTCTTTGATCGATGTTATTCATACGAACAAAAAGCTATATCTTGTGTTTG
AATATATCGATATGGACTTGCGAAAATTTATGGACTCGTTGGGAAATGACTCGATGCCTC
TCGCGCTCGTCAAGTCATACATTTGGCAGCTTTTGCAGGGCGTTGCGTTTTGCCATGCAC
AC

Cylin Ba dsRNA

ATGAATACTGAGCGAGAACAACTTCAAGCTCTCAACCGAGATGTGAACATCCAGGAGAG
CGGTCCCGTCAAGGCCGTGGTCAACGCCGAGAATGTCGAGGAGATGACATGGGAGGCG
GACGATAGTGGAGCGGACGCACTCGAAGCGCAGCTCAAGAGAATTGAGAGCTCGGATG
AGAAAGACGAGCGGTGGAGCAAAGACCACGTCGAGCAAATTTACGGCTACTTGCGCTTC
TTGGAGCATGCGAAGGAGGTCCGTCCGGACTTCCTCGCTCATCACGGGCAGAACGCAAC
GCCCAAG

Cyclin Bc dsRNA

TGGAGGCCCGTCGACTCAATCCTCGAAATCGCTTAGAAGGGGATGGATTAGTCAGTAAG
GGAATCGGTGGTATTGCCAAGCAGCGAACTGCATTGGGCGCTGTTCATGGAAGCAACAG
ACAGATAAAAGACGAGGCATCCAAGCCACATGTTAGAACCACCCGAGCTCAAATGCTCC
GATCAAAGATGGTGGACAATCAGCGAACGAAGCCAGCCGTCAAGTCTCAAAATCAGCCC
ATCAAGCGGAAGGCAGAAGCTAAAAAGGCTAAAATCGAAATTATCGAGGAAAAACCGC

References

DeLano WL. 2002. The PyMOL Molecular Graphics System. Delano Scientific, San Carlos.

Kumar S, Stecher G, Tamura K. 2016. MEGA7: Molecular evolutionary genetics analysis version 7.0 for bigger datasets. *Mol Biol Evol.* 33(7):1870-1874. doi:10.1093/molbev/msw054

Nie Y, Bellon-Echeverria I, Trowitzsch S, Bieniossek C, Berger I. 2014. Multiprotein complex production in insect cells by using polyproteins. *Methods Mol Biol.* 1091: 131-141. doi:10.1007/978-1-62703-691-7_8

Yang ZH. 2007. Paml 4: Phylogenetic analysis by maximum likelihood. *Mol Biol Evol*. 24(8):1586-1591. doi: 10.1093/molbev/msm088

Yang JY, Yan RX, Roy A, Xu D, Poisson J, Zhang Y. 2015. The I-TASSER Suite: Protein structure and function prediction. *Nat Methods* 12(1):7-8. doi: 10.1038/nmeth.3213

Zhang JZ, Rosenberg HF, Nei M. 1998. Positive darwinian selection after gene duplication in primate ribonuclease genes. *Proc Natl Acad Sci USA.* 95(7):3708-3713. doi.org/10.1073/pnas.95.7.3708

SUPPLEMENTARY TABLES

Table S1. CODEML analyses of selection among *Oikopleura CDK1* paralogs.

Branch site models	Site class	Proportion	ω Background	ω Foreground	Ln L	2 Δ L[a]	Positively selected sites[b]	
							NEB prob (ω >1)	BEB prob (ω >1)
	0	0.90440	0.01779	0.01779			N/A	N/A
Model A	1	0.01233	1.00000	1.00000	-9374.15			
null	2a	0.08215	0.01779	1.00000				
CDK1a	2b	0.00112	1.00000	1.00000				
Model A	0	0.90524	0.01850	0.01850		2,7649	25 T 0.970*	61 P 0.889
alternative	1	0.01253	1.00000	1.00000		(P = 0.0481)*	61 P 0.994**	85 M 0.962*
CDK1a	2a	0.08111	0.01850	**41.01038**	-9372.77		85 M 0.999**	200 K 0.836
	2b	0.00112	1.00000	**41.01038**			104 L 0.983*	
							178 S 0.981*	
							194 F 0.954*	
							200 K0.991**	
	0	0.91289	0.01717	0.01717			N/A	N/A
Model A	1	0.01462	1.00000	1.00000				
null	2a	0.07134	0.01717	1.00000	-9803.76			
CDK1b	2b	0.00114	1.00000	1.00000				
						7.5220 (P = 0.0030)**		
Model A	0	0.93943	0.01735	0.01735			21 G 0.989*	21 G 0.843
alternative	1	0.01303	1.00000	1.00000			68 D 0.977*	114 Q 0.970*
CDK1b	2a	0.04689	0.01735	**28.14003**	-9800.00		114 Q 0.998**	154 G 0.875
	2b	0.00065	1.00000	**28.14003**			154 G 0.992**	
Model A	0	0.92043	0.01727	0.01727			N/A	N/A
null	1	0.01183	1.00000	1.00000				
CDK1c	2a	0.06688	0.01727	1.00000				
	2b	0.00086	1.00000	1.00000	-9377.39			
						7,8597 (P = 0.0020)**		
Model A	0	0.92081	0.01831	0.01831			105 V 0.996**	183T 0.858
alternative	1	0.01200	1.00000	1.00000			107 S 1.000**	
CDK1c	2a	0.06632	0.01831	**999.00000**	-9373.46		117 V 0.992**	
	2b	0.00086	1.00000	**999.00000**			182 S 0.968*	
							183 T 1.000**	

Positively selected sites in CDK1a and b branches were identified by the empirical Bayes method implemented in PAML 4.1. [a]Branch-site model A is specified using the model 2 NSsites = 2, LRT = 2Δl, df = 1, with significances (*P<0.05; **P<0.01) determined by one-sided Chi-squared tests. [b]Only positively selected sites with naive empirical Bayes (NEB) or Bayes empirical Bayes (BEB) posterior probabilities (prior distribution estimated from the data) equal or greater to 95% (*), or 99% (**), are indicated. Numbering and residues based on human CDK1. N/A, not applicable.

Table S2. **Primer sequences for cyclin, CDK and reference genes**

Target transcript	Primer	Primer Sequence (5' to 3')
**Cyclins**		
Cyclin Ba/b	CCQ49F	GCAAGTTTACGGCTACTTGCGCTT
	CCQ50R	ATCCATGTACGCGACGGTGAGAAA
Cyclin Bc	CCQ55F	AAGGGAATCGGTGGTATTGCCAAG
	CCQ56R	AGCATTTGAGCTCGGGTGGTTCTA
**CDKs**		
CDK1a	CCQ393F	CTCATTTTCGAGTTTCTTTCGTGC
	CCQ394R	AGCCCTCCTTCCAGTCCTTG
	CCQ395F	GCCGAAATAGCAACGCTCAGA
	CCQ396R	TTTCCTAAGTCGCACATTGGC
CDK1b	CCQ401F	CGCAAGATCGTCAGAAGCAGA
	CCQ402R	CTCGACCAAAATCGTGATCAGATC
	CCQ403F	GCAATGGGTATCAAGAAACGTCTG
	CCQ404R	GCTGAAACGCCTTCCGCA
CDK1c	CCQ409F	ATCGACGACCAGAAAGCCG
	CCQ410R	GCCTGAACGGGATCTTGTAGAC
	CCQ411F	CGGCTGTACCTCACTACGACAAAG
	CCQ412R	GCAAAGCCACACGCACAGTA
CDK1d	CCQ397F	GGCATTCCGCCTACCAGTC
	CCQ398R	CTCATCGCCAAGCTCCTATCG
CDK1e	CCQ405F	GAATGACCAAAGCAAAGATGGC
	CCQ406R	TGGCGTCCCAAGCACTCTG
	CCQ407F	CGGATCGCTTTGATATACCTTCC
	CCQ408R	GCCTAATATGTGATGTCAGTGATAGAAG
CDK2	CCQ443F	TATGAGCCATCTCGGCGAATACCA
	CCQ444R	AGTTAGGCAGAACCTGTGCTCGAT
**Reference targets**		
RPL23	CCQ39F	CGACAGTCCAAGGCTTTCCGAAGA
	CCQ40R	CAGAGCCCTTCATTTCTCCCTTGT
EF1	CCQ43F	AGGTCATCCCTGAACTTAACGGCA
	CCQ44R	GGCAGATTTGATGGCAGCGTTGAT
**dsRNA**		
CDK1a	JIP120	TAATACGACTCACTATAGGGATGACGAAGGCGTACCAAGCACTA
	JIP121	TAATACGACTCACTATAGGGAGTCCTTGAAATTCTCGACGCCCT
CDK1b	JIP124	TAATACGACTCACTATAGGGTTTCGCCTTGGAGACGAGGAAGAA
	JIP125	TAATACGACTCACTATAGGGTGTCCGAGAAGAATTTCGGGAGCA
CDK1c	JIP128	TAATACGACTCACTATAGGGTTCTCGGAACACCGACCGAAGAAA
	JIP129	TAATACGACTCACTATAGGGAGAGGAAGATTCAAGATCGCCGC
CDK1d	JIP132	TAATACGACTCACTATAGGGAAGATATTCCGCATCCTCGGCACT
	JIP133	TAATACGACTCACTATAGGGACAGCGTTTGCTTTCGAGTTCTCC
CDK1e	JIP136	TAATACGACTCACTATAGGGTACGCATGTCCGGTTGATTGTTGG
	JIP137	TAATACGACTCACTATAGGGAGCAGGGACAGCTGGATATTCCT
CDK2	CDK2_F	TAATACGACTCACTATAGGGTAAAAACAGAGCAACAGGCAGA
	CDK2_R	TAATACGACTCACTATAGGGGTGTGCATGGCAAAACGCAACG
Cyclin Ba/b	Cyclin Ba/bRNAi_F	TAATACGACTCACTATAGGGATGAATACTGAGCGAGAACAACTTC

	Cyclin BaRNAi_R	TAATACGACTCACTATAGGGCTTGGGCGTTGCGTTCTGCCCGTG ^
Cyclin Bc	XM70	TAATACGACTCACTATAGGGTGGAGGCCCGTCGACTC
	XM71	TAATACGACTCACTATAGGGGCGGTTTTTCCTCGATAATTTCGA

SUPPLEMENTARY FIGURES

Figure S1. Intron-exon structures of *O. dioica* and *O. albicans* CDK1 genes. Maximum likelihood inference of selected chordate CDK1 proteins using CDK1 kinase domains with posterior probabilities indicated at nodes. Intron phases in coding regions are indicated as 0, 1 and 2. Conserved regions within *O. dioica* and *O. albicans* CDK1 sequences are indicated as light blue bars. The region encoding the PSTAIRE helix has been split by a phase 2 intron in orthologs of *Oikopleura CDK1b* and *c*.

Figure S2. Substitutions in odCDK1 paralogs at evolutionarily conserved metazoan CDK1 residues. (*A*) Conserved metazoan CDK1 residues identified by alignment of CDK1 sequences from 15 metazoan phyla. The budding yeast cdc28 ortholog is also included in the alignment. Substitutions in *Oikopleura* CDK1 sequences that occur at sites conserved in other metazoan CDK1s were labeled with blue squares and mapped to structural elements of the active CDK2-cyclin A1 complex crystalized with ADP, substrate peptide and MgF_3^-, a mimic for the γ-phosphate of ATP in the transient state (PDB ID: 3QHR). (*B*) Protein kinase structural elements are shown in white and the residues that form the regulatory and catalytic spine are shown as space-filling dots. The upper right panel shows a magnified ATP binding region and the lower panel shows a zoomed-in view of the corresponding surface-filled model. Numbering is according to human CDK2. Functional motifs are differently coloured. ADP is shown as stick model with MgF_3^-, a mimic for the γ phosphate group indicated as a yellow sphere in all panels. Mg^{2+}(1) and Mg^{2+}(2) are shown as white spheres in the upper right panel. Residues critical for catalysis are conserved in *Oikopleura* CDK1 paralogs including: the Mg^{2+}-binding loop, the catalytic loop, and the R- and C-spines. The residues associated with T160 (R50, R126, R150) are also conserved. The residues in *Oikopleura* CDK1s (adenine region, sugar region, phosphate binding region) that interact directly with ATP are conserved, whereas residues that enclose and modulate the affinity to ATP (hinge and solvent accessible region) are diversified. None of the CDK1 outparalogs have the same ATP binding sites. Again, the residues in the activation loop that direct contact with P0 and P+3 are conserved in *Oikopleura* CDK1 paralogs, but residues that modulate the conformation of the activation loop are diversified in *Oikopleura* CDK1 outparalogs. None of the *Oikopleura* CDK1 outparalogs has the same activation loop sequence. Bottom right: residues in the hinge and solvent accessible region of human (hs) CDK2, CDK1, and 3 *O. dioica* (od) CDK1 paralogs are compared. (*C*) Schematic diagram of the protein kinase catalytic mechanism. The reaction proceeds from the enzyme/substrate complex

through the transition state (center) to the enzyme/product complex. Residue numbers are based on human CDK2. The OH group of the protein substrate is aligned so that the electron pair on the oxygen are in-line with the γ-phosphate (arrow) and the βγ-bridging oxygen (curved arrow) of the bound ATP.

Figure S3. Mapping of *Oikopleura* CDK1 substitution sites to the human CDK1 (green)-cyclin B1 (blue) structure, indicating positive selection sites and conserved determinants of specificity. (*A*) Substitutions in *Oikopleura* CDK1 sequences that occur at sites conserved in other metazoan CDK1s are indicated in blue. Evolutionarily conserved determinants of substrate specificity that are modified in *Oikopleura* CDK1 paralogs are indicated in black. The conserved PSTAIRE helix, which is modified in *Oikopleura,* is indicated in red. Blue spheres indicate residues that have undergone positive selection as identified by PAML 4.1 (sites with naive empirical Bayes posterior probabilities equal or greater to 95% and also Bayes empirical Bayes posterior probabilities greater to 87%). The black box outlines the T-loop that is enlarged in (*B*). The red box outlines the PSTAIRE helix and its interface with Cyclin B that is enlarged in (*C*). (*B*) CDK1-specific β turn formed at the tip of the activation segment when CDK1 binds cyclin B1. Residues involved in the β turn are A152, F153, G154, I155 (numbering based on human CDK1). Hydrogen bonds formed between main chains are labeled with black dashed lines. **c**, CDK1-specific hydrogen bonds formed between the side chain of S53 in the PSTAIRE helix and residues L296, G297 of cyclin B1. Colour coding in (*B*) and (*C*) as in (*A*).

Figure S4. CDK1/2 L167/L166 contribute to affinity toward the substrate P-2 residue. (*A*) Active human CDK2-Cyclin A1 complex crystalized with ADP, MgF_3^-, a mimic for the γ-phosphate of ATP in the transient state (PDB ID: 3QHR) and substrate peptide. Residues in the activation segment of CDK2 that contact the substrate P-2 residue are coloured in magenta. or (*B*) magentas spheres. The residue numbering corresponds to the human CDK2 sequence in (*A*) and the human CDK1 sequence in (*B*). *Oikopelura* CDK1a and b paralogs share an L167M substitution. *Oikopleura* substitutions in conserved metazoan CDK1 sites are labeled in blue, the activation segment in magenta, the substrate in orange, the catalytic loop in cyan, and the PSTAIRE helix in red.

Figure S5. CDK1c and e are dispensable for *Oikopleura* embryogenesis. (*A*) Left panel: significant knockdown *CDK1*c in oocytes spawned from ovaries that had been injected with dsRNA against *odCDK1c* at D4 (n1 = 3, n2 = 3). Right panel: no significant difference in developmental outcome between *CDK1c* deficient and wild type zygotes (n1 =3, n2 = 3). The top legend for this panel also applies to the right panels in (*B*) and (*C*). (*B*) Left panel: significant knockdown of *CDK1e* in oocytes spawned from ovaries that had been injected with dsRNA against *odCDK1e* at D4 (n1 = 3, n2 = 3). Right panel: no significant difference in developmental outcome between *CDK1e* deficient and wild type zygotes (n1 = 3, n2 = 3). (*C*) Upper left panel: significant) knockdown *CDK1c and CDK1e* in oocytes spawned from ovaries that had been injected with dsRNA against *odCDK1c* and *CDK1e* at D4

(upper left panel) (n1 = 3, n2 = 3). Upper right panel: no significant difference in developmental outcome between *CDK1c* + *CDK1e* deficient and wild type zygotes (upper right panel) (n1 = 3, n2 = 3). Where indicated, ***P<0.001, **P = <0.01, *P<0.05. Bottom panel: Western blot showing knockdown of CDK1c in *CDK1c* RNAi and *CDK1c + e* RNAi oocytes.

Figure S6. Cellular localization of CDK1 paralogs during interphase and M-phase. Immunostaining in tadpoles derived from oocytes injected with CDK1-GFP capped Mrna. Antibodies were against the PSTAIRE helix of CDK2 or anti-eGFP antibodies targeting odCDK1a, b or d-eGFP fusion constructs. H3-pS28 antibody staining on centromeres. CDK2 disappeared on chromatin during early prophase, CDK1a initiated on chromatin during interphase and disappeared prior to metaphase. CDK1b was absent from chromatin during interphase and became intense on chromatin during the prophase to pro-metaphase, before declining on metaphase chromatin. CDK1d exhibited exclusion from chromatin until prophase when there was diffuse staining on chromatin, which largely disappeared in metaphase. Scale bars: 5 µm.

Figure S7. *Oikopleura* CDK1d:Cyclin Ba/b-specific substitutions in the interfacial region. (*A*) Alignment of amino acids in Cyclin B homologs that mediate the CDK1:Cyclin B interface, as identified using CCP4 CONTACT (PDB ID: 4YC3). (*B*) Human CDK1:Cyclin B1 structure (PDB ID: 4YC3) showing conserved contacts between the Cyclin B1 N-terminal Y170 and the CDK1 C-lobe hydrophobic pocket. Cyclin B substitutions at the Y170 site were present in odCyclin Ba/b, not in Bc. The salt bridge formed between CycB1 E173 and CDK1 K279 is conserved in metazoans and plants, but this pair of residues has been modified in odCDK1d (or e):CycBa/b pairings. Sequences were from *Oikopleura dioica, Homo sapiens, Strongylocentrotus purpuratus, Marthasterias glacialis, Drosophila melanogaster, Hydra viridissima, Saccharomyces cerevisiae,* and *Chlamydomonas reinhardtii*.
